# Estimating lifetime and 10-year risk of lung cancer^☆^

**DOI:** 10.1016/j.pmedr.2018.06.010

**Published:** 2018-06-18

**Authors:** Christina Bruder, Jean-Luc Bulliard, Simon Germann, Isabelle Konzelmann, Murielle Bochud, Magali Leyvraz, Arnaud Chiolero

**Affiliations:** a Institute of Social and Preventive Medicine (IUMSP), Lausanne University Hospital, Lausanne, Switzerland; b Observatoire Valaisan de la santé (OVS), Sion, Switzerland; c Institute of Primary Health Care (BIHAM), University of Bern, Switzerland; d Department of Epidemiology, Biostatistics and Occupational Health, McGill University, Montréal, Canada

**Keywords:** Lung cancer, Risk communication, Lifetime risk, Public health indicator, Switzerland

## Abstract

Lung cancer is the commonest cancer worldwide. Mortality and incidence rates are traditionally used to assess cancer burden and as public health indicators. However, these metrics are difficult to interpret at an individual level. Providing the lifetime and 10-year risks of cancer could improve risk communication. Our aim was to estimate current lifetime and 10-year risks of lung cancer by smoking status and changes in these risks between 1995 and 2013 in a Swiss population. We used all lung cancer cases recorded between 1995 and 2013 by two population-based cancer registries in the contiguous cantons of Vaud and Valais, in Western Switzerland. We estimated sex-specific lifetime risk and 10-year risk of lung cancer using the current probability method, accounting for competing risk of death. Estimates were also provided by smoking status. Between 1995 and 2013, 9623 cases of lung cancer were recorded. During this period, the lifetime risk decreased in men from 7.1% to 6.7% and increased in women from 2.5% to 4.1%. In both sexes, the 10-year risk of lung cancer increased with age until the age of 60–70 and decreased thereafter. Difference in the cumulative risk between current, former, and never smokers were very large and reported in user-friendly charts to ease risk communication. These lifetime and 10-year risk estimates could be used systematically as public health indicators. Regularly updating risk estimations are necessary for conditions like lung cancer whose incidence has changed substantially.

## Highlights

• Providing the lifetime and cumulative 10-year risks of cancer by smoking status could improve risk communication and serve as public health indicators.
• These indicators can be estimated with cancer registry data and smoking data from surveys using the current probability method.
• In these regions of Switzerland, between 1995 and 2013, the lifetime risk of lung cancer decreased in men from 7.1% to 6.7% and increased in women from 2.5% to 4.1%.
• Difference in the cumulative risk between current, former, and never smokers were very large.

## 1 Introduction

In 2012, lung cancer was the commonest diagnosed cancer in men worldwide and the third most frequently diagnosed cancer in women, after breast and colorectal cancer (Torre et al., 2015). In Switzerland, between 2008 and 2012, it was the leading cause of cancer death in men and the second cause of cancer death in women, after breast cancer (Arndt et al., 2016). Because smoking is the major cause of lung cancer (Janssen-Heijnen and Coebergh, 2001), trends in lung cancer incidence are closely following trends in smoking habits in the population, with a latency time of about 30 years (Oberli et al., 2016). In Switzerland, smoking prevalence peaked in the 1950s in men and in the 1970s among women (Oberli et al., 2016). Hence, lung cancer incidence reached in men a peak in the 1980s and decreased thereafter (Janssen-Heijnen and Coebergh, 2003; Levi et al., 1997). Among women, the incidence has increased at least since the 1970s (Levi et al., 1997) and, apparently, has not reached a peak yet (Rapiti et al., 2014).

Because cancers are feared diseases, an adequate risk communication about the individual chance of developing cancer is important (Schwartz et al., 1999; Gigerenzer et al., 2010; Kurz-Milcke et al., 2008). To assess the burden of cancer, mortality and incidence are frequently used. They are also standard public health indicators. However, these epidemiological and population level metrics are not simple to use for risk communication. Indeed, it might be difficult for lay persons to grasp one's own risk of having e.g. a lung cancer through age-standardized or crude rates of incidence or mortality (Sasieni and Adams, 1999). To improve health risk communication, we can estimate the lifetime risk, which is the cumulative risk of being affected by a disease during the existence (Germann et al., 2016). Lifetime risk appears to be an informative and easily understood measure of the risk of disease in individuals (Narayan et al., 2003). As many people are interested in their own risk of disease in the near future (Woloshin et al., 2008a), the cumulative 10-year risk, i.e. the probability, in percentage, for a given individual of developing lung cancer in the next 10 years, could be another interesting metric to help patients and health professionals for health decision making (screening or change in health behavior) and to facilitate the discussions about cancer risk. Importantly, these risks have to be regularly updated, following changing incidence across time.

The lifetime risk of cancer has been estimated and is publicly available in several countries, such as Canada or the United States. To our knowledge, the 10-years cumulative risk is not systematically computed by any country worldwide. Our goal was therefore to estimate the sex-specific lifetime risk and 10-year risk of lung cancer in a Swiss population, by smoking status, and assess changes in these risks between 1995 and 2013. Further, we provided sex-specific user-friendly tables and graphical tools for risk communication about lung cancer.

## 2 Material and methods

### 2.1 Study design

We conducted a population-based temporal trend study of the risk of lung cancer in two regions of Western Switzerland (cantons of Vaud and Valais) between 1995 and 2013. The total number of inhabitants in the cantons of Vaud and Valais is 1,123,998 corresponding to 13% of the total population of Switzerland.

### 2.2 Data source

In Switzerland, data on all new cancer cases are collected, documented and recorded by population-based regional cancer registries. The National Institute for Cancer Epidemiology and Registration (NICER) compiles and aggregates this data (www.nicer.org). Quality control procedures are based on the guidelines from the European Network of Cancer Registries (Working Group of the International Association of Cancer R, 2005). Variables routinely harvested by Swiss cancer registries include notably the birth date, sex, status at last follow-up (alive, dead or left canton) and date of last follow-up of the patient, as well as for each tumor its date of diagnosis (termed date of incidence), localization, histological type, malignancy, stage TNM and size.

We used data from the Vaud Cancer Registry (Registre Vaudois des tumeurs; RVT) at the Institut Universitaire de Médecine Sociale et Préventive (IUMSP; www.iumsp.ch) and the Valais Cancer Registry (Registre Valaisan des tumeurs; RVsT) at the Observatoire Valaisan de la Santé (OVS; www.ovs.ch). These registries collect data on all new (incident) cases of cancer diagnosed in the cantons of Vaud and Valais, since 1974 and 1989 respectively. Data on all cases of lung cancer recorded between 1995 and 2013 were used for this study. A recent evaluation of the completeness of cancer case ascertainment in Switzerland, including the Vaud and Valais Cancer Registries, indicated a high completeness for most cancer types, including lung cancer (Lorez et al., 2017). Annual mortality records were provided by the Federal Statistical Office. Death rates of lung cancer were extracted from these records. Information on smoking prevalence were obtained through Suchmonitoring Schweiz (www.suchtmonitoring.ch).

### 2.3 Statistical analyzes

We analyzed trend data across 4 time periods, i.e., 1995–1998, 1999–2003, 2004–2008, and 2009–2013. Annual lung cancer incidence and mortality rates, stratified by sex, were computed. Rates were age-standardized to the European standard population (Curtin and Klein, 1995).

The current probability method was used to estimate the lifetime risk (Sasieni et al., 2011). This method allows the estimation of cumulative risk of any condition throughout lifetime, using the population current incidence and mortality at each age. It accounts for the risk of being affected for the first time by the condition at each age, and for the competitive risk of dying (and not reaching that age) (Germann et al., 2016; Sasieni et al., 2011). With data containing only primary tumors, the current probability method is considered as the “gold standard” to estimate the lifetime risk of any conditions during a given span of life (Sasieni et al., 2011). To use this method, we estimated the number of first cancer cases and the number of cancer-free individuals (by definition, not recorded in cancer registries). We also estimated the rates of cancer (incidence) and of all-cause mortality (Ahmad et al., 2015). We assumed that, within each time period considered, the incidence and mortality rates of lung cancer were constant over time for each 5-year age group. For each year of life, we calculated the risk (in %) of lung cancer. The cumulative lifetime risk (in %) was computed for men and women and for each time-period by the sum of these risks over the lifespan.

We created a 10-year lung cancer cumulative risk table and chart, inspired by Woloshin et al. (Woloshin et al., 2008b). To estimate the 10-year cumulative risk of lung cancer at age A, we computed the difference between the cumulative risk until age A + 10 years and the cumulative risk until age A. For example, the cumulative 10-year risk since the age of 40 years old (that is, between the age of 40 and 49) is the difference between the cumulative risk of having a lung cancer at age 50 minus the cumulative risk at age 40. The 10-year risk was estimated at age 20, 30, 40, 50, 60, 70, 80, and 90. Since the risk of lung cancer differs by sex, we built separate tables and charts for men and women.

We estimated the lifetime and 10-year risks by smoking status indirectly (Woloshin et al., 2008b; Becher et al., 2018). First, we used the sex-, age- and time-period specific prevalence of smoking in Switzerland (Supplemental Table S1). Second, we used the relative risk (RR) of lung cancer associated with current and former smoking compared to never smoking, derived from a recent systematic review (Lee et al., 2012) (i.e., RR = 6.57 for current smokers below 60 years of age, 9.62 for current smokers between 60 and 69, 9.07 for current smokers 70 years old or more, and 4.30 for former smokers of all ages). For each year of life, we calculated the risk (in %) of lung cancer by smoking status with the following formulas:

• $${\text{Risk}}_{\text{never smokers}}={\text{risk}}_{\text{whole population}}/\left({\text{Prevalence}}_{\text{never smokers}}+{\text{Prevalence}}_{\text{current smokers}}\times {\mathrm{RR}}_{\text{current smokers}}+{\text{Prevalence}}_{\text{former smokers}}\times {\mathrm{RR}}_{\text{former smokers}}\right)$$
• $${\text{Risk}}_{\text{current smokers}}={\text{risk}}_{\text{never smokers}}\times {\mathrm{RR}}_{\text{current smokers}}$$
• $${\text{Risk}}_{\text{former smokers}}={\text{risk}}_{\text{never smokers}}\times {\mathrm{RR}}_{\text{former smokers}}$$

The cumulative lifetime and 10-year risks were computed for current, former, and never smokers by the sum of these risks over specific lifespans.

### 2.4 Ethics

Anonymous health related personal data with no possibility to identify individuals and recorded by cancer registries for research activities were used. There was no threat to patient confidentiality. According to the Swiss Human Research Act (Humanforschungsgesetz HFG), no ethical approval was needed for such analyses.

## 3 Results

Between 1995 and 2013, 9623 lung cancer cases were diagnosed in Vaud (69% of cases) and Valais (31% of cases), of which 6317 occurred in men (66% of cases) and 3306 in women (34%) (Table 1 & Supplemental Table S2). The standardized incidence rate of lung cancer decreased by 27% in men (from 73/100,000 to 53/100,000 inhabitants) and increased by 41% in women (from 22/100,000 to 31/100,000 inhabitants) (Table 1). There were 7152 deaths from lung cancer in Vaud (70% of deaths) and Valais (30%) between 1995 and 2013. During the same time period, the standardized mortality rate of lung cancer decreased in men by 28% and increased in women by 19% (Table 1). Mortality rates from lung cancer appear however to have stabilized in women from 1999 to 2003 onwards. Between 1995 and 2013, the prevalence of smoking decreased in men more than in women.

**Table 1:** Standardized (European population) incidence and mortality rates per 100,000 inhabitants of lung cancer, by sex, per period, between 1995 and 2013, Vaud and Valais, Switzerland (Source: Vaud and Valais Cancer Registries) and prevalence of current, former, and never smokers between 1995 and 2013 in Switzerland (Source: Suchtmonitoring Schweiz). N: number; Δ: absolute and relative (in %) difference in incidence and mortality rates between time-periods.

	Sex	1995–1998	1999–2003	2004–2008	2009–2013	Δ 1995–1998 to 2009–2013
N cases	Men	1299	1685	1677	1656	
	Women	481	770	937	1118	
Incidence rate/100,000	Men	73	69	62	53	−20 (−27%)
	Women	22	27	29	31	+9 (+41%)
N deaths	Men	937	1305	1310	1254	
	Women	370	573	668	735	
Mortality rate/100,000	Men	54	53	48	39	−15 (−28%)
	Women	16	19	20	19	+3 (+19%)
Prevalence of current smokers	Men	39.1%	36.0%	32.3%	32.4%	−6.7%
	Women	27.8%	25.5%	23.6%	24.2%	−3.6%
Prevalence of former smokers	Men	23.3%	23.9%	24.3%	24.6%	+1.3%
	Women	15.5%	16.2%	18.2%	18.6%	+3.1%
Prevalence of never smokers	Men	37.6%	40.2%	43.4%	43.0%	+5.4%
	Women	56.8%	58.4%	58.2%	57.3%	+0.5%

**Fig. 1:**
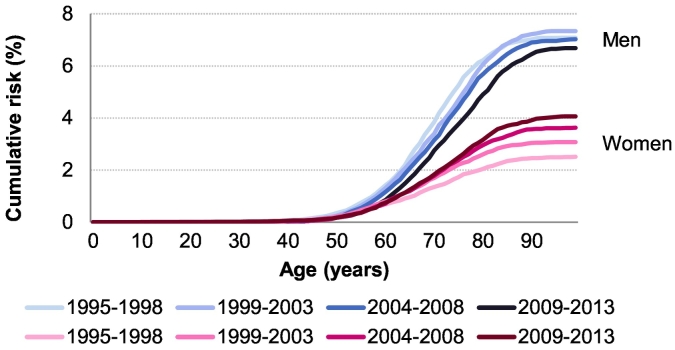
Cumulative risk (in absolute %) of lung cancer incidence, by sex, across ages, per time-period, between 1995 and 2013, in Vaud and Valais, Switzerland. (Source: Vaud and Valais Cancer Registries). The darker the colour, the more recent the time-period.

Fig. 1 shows the cumulative risk (in absolute %) of lung cancer in men and women across age, per time period, between 1995 and 2013. The darker the colour, the more recent the time-period. Across all ages, the cumulative risk decreased in men between 1995 and 1998 and 2009–2013 whereas it increased in women. Although the cumulative risk remained higher among men than women, the sex difference in cumulative risk decreased with time.

The lifetime risk decreased in men from 7.1% to 6.7% (with a maximum of 7.3% in 1999–2003) while it increased regularly among women from 2.5% to 4.1%, between 1995 and 1998 and 2009–2013, respectively (see Supplemental Fig. S1). The difference in lifetime risk between men and women decreased with time.

**Fig. 2:**
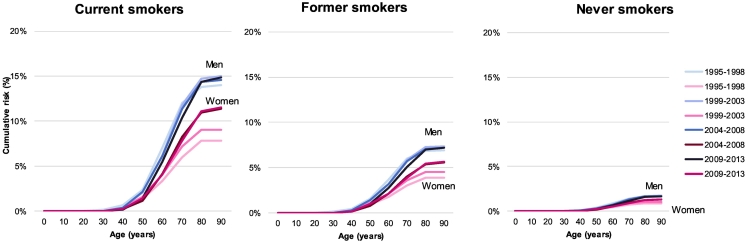
Cumulative risk (in %) of lung cancer, by smoking status (A: current smokers, B: former smokers, C: never smokers), by sex, age, and time-period, between 1995 and 2013, Vaud and Valais, Switzerland.

Source: Vaud and Valais Cancer Registries, Switzerland

**Fig. 3:**
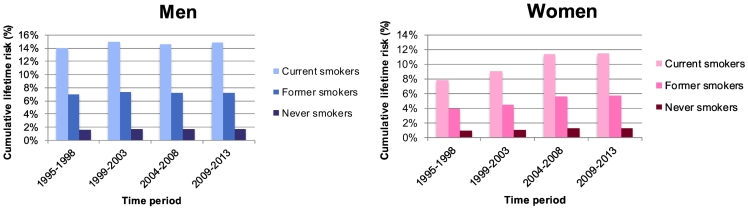
Lifetime risk (in %) of lung cancer, by smoking status (dark blue/pink: current smoker, medium blue/pink: former smoker, light blue/pink: never smoker), by sex and time-period, between 1995 and 2013, Vaud and Valais, Switzerland.

Source: Vaud and Valais Cancer Registries, Switzerland

The cumulative risk and lifetime risk of lung cancer, by sex, ages, and smoking status are reported in Fig. 2, Fig. 3. The difference in cumulative risk was very large between current, former and non-smokers, in both sexes and across all time-periods.

**Fig. 4:**
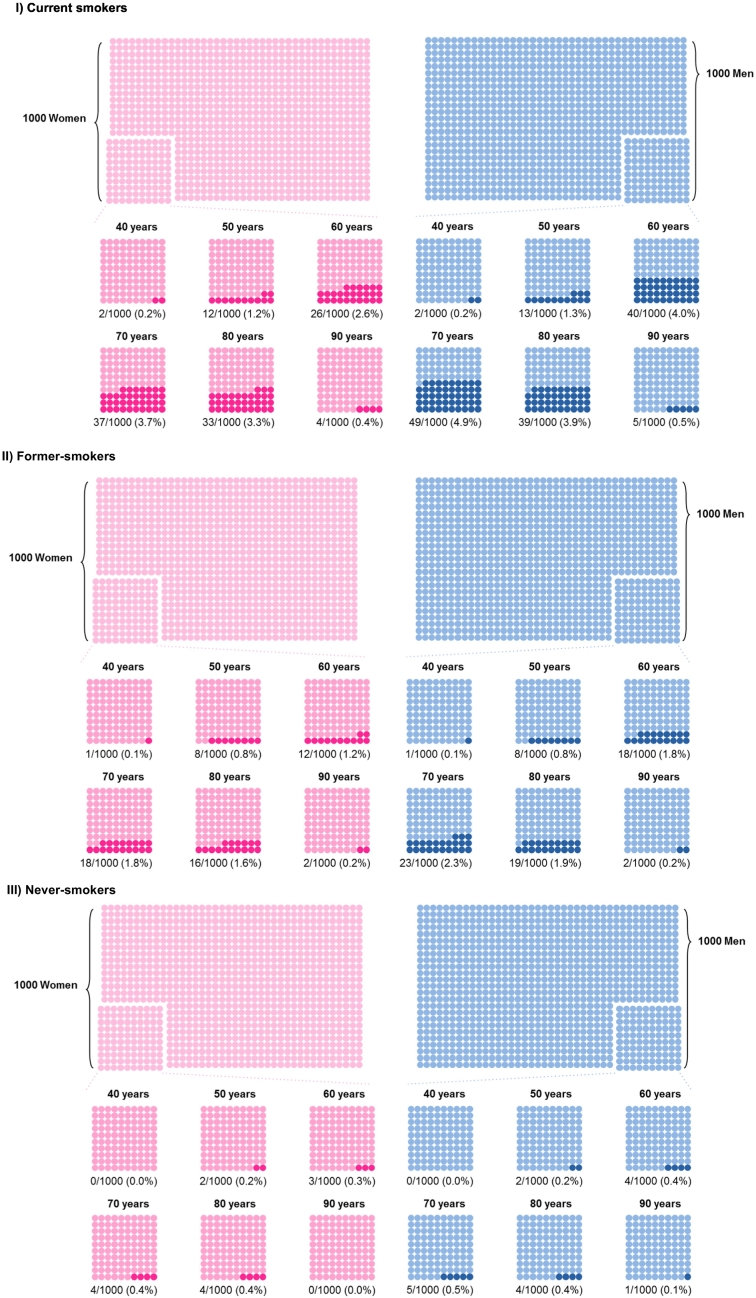
This chart represents the 10-year risk (in absolute %) of lung cancer, by smoking status, 10-years age group, and sex, for the most recent period (2009–2013), Vaud and Valais, Switzerland. Each dot corresponds to one individual.

Source: Vaud and Valais Cancer Registries

The 10-year risk (in absolute %) of lung cancer, by age group and time-period in men and women, between 1995 and 2013 are shown in Supplemental Table S3. Using estimates for the time period 2009–2013, a 10-year risk chart was designed to facilitate the representation of the individual risk of developing lung cancer over the next 10 years in men and women between 40 and 90 years of age, according to smoking status (Fig. 4). In both sexes, the 10-year risk of lung cancer increased with age from age 40 to 70 and decreased thereafter.

## 4 Discussion

Between 1995 and 2013, the lifetime risk of lung cancer in Switzerland has slightly decreased in men from 7.1% to 6.7% while it increased in women from 2.5% to 4.1%. The difference in lifetime risk has consequently decreased between men and women with time. The 10-year cumulative risk of lung cancer has decreased in men at all ages, excepted in men over 80 years of age in whom the risk increased. Among women, the 10-year cumulative risk increased from age 50 years onwards. Difference in the cumulative risk between current, former, and never smokers were very large and reported in user-friendly charts to ease risk communication. Updating risk estimations are necessary for adequate communication on conditions like lung cancer whose incidence has changed substantially. Further, these lifetime and 10 years cumulative risk estimates could be used systematically as public health indicators.

To our knowledge, no previous study has estimated the lifetime risk and 10-year cumulative risk of lung cancer in Switzerland. While several studies have estimated the lifetime or period-specific risk of being diagnosed with any cancer, lifetime risk of lung cancer has been rarely estimated in other countries. In Scotland, between 2001 and 2005, a study estimated the lung cancer lifetime risk to be 8.8% in men and 6.5% in women (Sasieni et al., 2011). In Canada, the lifetime risk of developing lung cancer was 8.1% in men and 4.7% in women between 1996 and 2000 (Chen, 2013). These lifetime risks are close, albeit slightly higher, than our estimations. However, such comparisons are of limited relevancy since these estimates strongly depend on the history of smoking behaviors in the compared populations.

Several strengths of our study deserve to be noted. First, it is based on high quality population data from two cancer registries recording all cases of cancers in patients living in a region of Western Switzerland. It allows us estimating reliably the incidence and mortality rates, as well as the cumulative risk of lung cancer. Second, we used the current probability method described as the gold standard for estimating cumulative and lifetime risk with data containing only the first primary cancer (Sasieni et al., 2011). This method requires complete information on the number of deaths in the population, to account for mortality competitive risk. Of note, this method could easily be applied for other cancers and to other countries. Third, we provide up-to-date user-friendly 10-year risk charts that could be used for risk communication about lung cancer.

Our study has also some limitations. It is restricted to two cantons of Switzerland. Lung cancer standardized incidence rate decreased in Switzerland between 1995 and 2013 from 66.9 to 50.1/100,000 and increased in women during the same time period from 18.2 to 27.7/100,000 (NICER: Foundation National Institute for Cancer Epidemiology and Registration, 2013). These national incidence are close to the incidence observed in Vaud and Valais, and our finding could to some extend by valid for the whole country. However, our goal was not to have accurate estimates for the whole country but to compute (10-year and lifetime) cumulative risk of lung cancer and provide user-friendly charts to ease risk communication. Another limitation is that we had to estimate the risk by smoking status indirectly, using relative risk of lung cancer (Woloshin et al., 2008b) and smoking prevalence from other sources. This approach might have under- or over-estimate the difference in risk between current, former and never smokers. While we developed user-friendly figures to ease the risk communication, we did no test if they improve the risk understanding among patients. Finally, the current probability method applies the sex- and age-specific current cancer incidence and mortality rate as if they were valid for a current cohort of ageing men or women; this would be true only if these rates remained stable over time.

Information is central in health decision making for patients and health professionals, as well as for policy makers (Woloshin and Schwartz, 1999). To take some health decision (e.g., to be screened for a cancer or to change health behaviors like smoking), patients and physicians need to be informed about the risk of developing disease over a defined period of time. Providing understandable quantitative information to patients and physicians can be challenging because information needs and understanding in numbers (health literacy) can vary a lot between individuals (Woloshin and Schwartz, 1999). The commonly used incidence and mortality rates are not adequate to grasp the risk of developing a cancer over a defined period of time. Lifetime and 10-year risks provide absolute risk estimates and are more intuitive metrics that could be more easily understood (Lloyd-Jones et al., 2006). Furthermore, presenting a user-friendly 10-year risk chart eases the person to realize the magnitude of health risk faced. Using that kind of chart during medical visits could facilitate the information sharing between doctors and patients, and help informed health decision making. It would be also useful for public health policy makers and health professionals to have health indicators based on these risk metrics. According to Woloshin et al. “useful messages about health risks should address two questions: how big is my risk and how does this risk compare with other risks” (Woloshin et al., 2008b). It would be therefore useful to develop similar charts for other cancers and diseases in order to put the risk in context and facilitate the representation of the magnitude of one's individual risk.

Importantly, risk of diseases need to be frequently updated in order to convey correct and reliable information to patients and doctors. For most diseases, risk charts have indeed a local or regional value, depending on the risk level in a given population and can be outdated, if the risk of disease changes promptly across time (Agarwal et al., 2009). When estimating lifetime risk of disease, it is also necessary to account for changes in life expectancy and in the competing risks of death. It is therefore necessary to update these risk charts, especially for conditions like lung cancer whose incidence has changed substantially following population-level changes in smoking behaviors.

## 5 Conclusion

Because mortality and incidence rates - both traditionally used to assess cancer burden at a population level - might be difficult to interpret at an individual level, we propose life-time and 10-year risks to be estimated systematically for all major cancers and used as public health indicators. Charts showing the huge difference in cumulative risk in current, former and never smokers could be used to help current smokers realize the benefit to quit smoking.

## Conflict of interest

None.

## Appendix A Supplementary data

Supplementary material Image 1
