# Supplementary material for: Estimating lifetime and 10-year risk of lung cancer
Source: Prev Med Rep. 2018 Jun 18;11:125–30. doi: 10.1016/j.pmedr.2018.06.010 (PMC6010924; doi:10.1016/j.pmedr.2018.06.010)
Supplement: Supplementary file 1 — Supplementary material [file mmc1.docx]

**Supplemental Table S1.** Prevalence of current smokers, formers smokers and never smokers in Switzerland by age, sex, and time-period, according to Suchmonitoring Schweiz (<http://www.suchtmonitoring.ch>, accessed on 20.02.2018).

| **Current smokers** | **Sex** | **15-24 y** | **25-34 y** | **35-44 y** | **45-54 y** | **55-64 y** | **65-74 y** | **75+ y** | **total** |
| --- | --- | --- | --- | --- | --- | --- | --- | --- | --- |
| 1995-1998 | Men | 46.6% | 45.2% | 44.3% | 40.6% | 30.8% | 24.5% | 19.9% | 39.1% |
| 1995-1998 | Women | 40.5% | 35.7% | 35.9% | 28.9% | 20.7% | 11.4% | 6.6% | 27.8% |
| 1999-2003 | Men | 39.9% | 42.2% | 39.9% | 39.8% | 30.3% | 24.6% | 15.1% | 36.0% |
| 1999-2003 | Women | 34.8% | 30.0% | 33.5% | 28.4% | 20.7% | 12.8% | 6.1% | 25.5% |
| 2004-2008 | Men | 36.6% | 43.2% | 32.3% | 32.8% | 30.8% | 23.3% | 11.2% | 32.3% |
| 2004-2008 | Women | 30.5% | 30.3% | 26.9% | 29.6% | 19.6% | 10.7% | 5.7% | 23.6% |
| 2009-2013 | Men | 39.6% | 43.3% | 38.8% | 32.6% | 28.0% | 19.0% | 9.7% | 32.4% |
| 2009-2013 | Women | 32.4% | 30.8% | 25.7% | 25.7% | 26.2% | 16.4% | 7.6% | 24.2% |
| **Former smokers** | **Sex** | **15-24 y** | **25-34 y** | **35-44 y** | **45-54 y** | **55-64 y** | **65-74 y** | **75+ y** | **total** |
| 1995-1998 | Men | 3.2% | 9.8% | 20.1% | 30.2% | 37.2% | 43.8% | 44.4% | 23.3% |
| 1995-1998 | Women | 5.0% | 13.0% | 19.7% | 21.6% | 17.7% | 14.6% | 14.2% | 15.5% |
| 1999-2003 | Men | 4.3% | 12.3% | 17.8% | 30.1% | 38.9% | 42.0% | 44.5% | 23.9% |
| 1999-2003 | Women | 5.4% | 12.4% | 18.5% | 22.5% | 21.2% | 17.0% | 14.5% | 16.2% |
| 2004-2008 | Men | 3.7% | 12.6% | 19.7% | 28.5% | 35.3% | 42.8% | 54.0% | 24.3% |
| 2004-2008 | Women | 5.1% | 13.9% | 18.1% | 24.7% | 25.6% | 23.7% | 16.9% | 18.2% |
| 2009-2013 | Men | 3.4% | 13.4% | 19.6% | 23.4% | 34.7% | 45.5% | 50.7% | 24.6% |
| 2009-2013 | Women | 3.4% | 17.4% | 17.2% | 23.0% | 24.3% | 25.3% | 17.8% | 18.6% |
| **Never smokers** | **Sex** | **15-24 y** | **25-34 y** | **35-44 y** | **45-54 y** | **55-64 y** | **65-74 y** | **75+ y** | **total** |
| 1995-1998 | Men | 50.2% | 45.1% | 35.5% | 29.2% | 32.0% | 31.7% | 35.7% | 37.6% |
| 1995-1998 | Women | 54.4% | 51.2% | 44.4% | 49.5% | 61.5% | 74.0% | 79.2% | 56.8% |
| 1999-2003 | Men | 55.8% | 45.5% | 42.3% | 30.1% | 30.8% | 33.4% | 40.4% | 40.2% |
| 1999-2003 | Women | 59.9% | 57.6% | 48.0% | 49.1% | 58.1% | 70.2% | 79.4% | 58.4% |
| 2004-2008 | Men | 59.8% | 44.2% | 48.0% | 38.6% | 33.8% | 33.9% | 34.8% | 43.4% |
| 2004-2008 | Women | 64.4% | 55.8% | 55.0% | 45.7% | 54.9% | 65.7% | 77.4% | 58.2% |
| 2009-2013 | Men | 57.1% | 43.3% | 41.6% | 44.0% | 37.2% | 35.5% | 39.7% | 43.0% |
| 2009-2013 | Women | 64.2% | 51.8% | 57.1% | 51.3% | 49.5% | 58.3% | 74.5% | 57.3% |

**Supplemental Table S2:** Standardized (European population) incidence and mortality rates per 100’000 inhabitants of lung cancer by sex, region, and time-period, between 1995 and 2013, Vaud and Valais, Switzerland (Source: Vaud and Valais Cancer Registries, Switzerland). Δ: absolute difference in incidence and mortality rates between time-periods.

|  | **Region** | **Sex** | **1995-1998** | **1999-2003** | **2004-2008** | **2009-2013** | **Δ 1995-1998**  **to 2009-2013** |
| --- | --- | --- | --- | --- | --- | --- | --- |
|  |  |  |  |  |  |  |  |
| **Incidence** | Vaud | Men | 74 | 69 | 62 | 53 | - 21 |
|  |  | Women | 23 | 28 | 32 | 30 | + 7 |
|  | Valais | Men | 72 | 69 | 62 | 53 | - 19 |
|  |  | Women | 20 | 26 | 24 | 32 | + 12 |
| **Mortality** | Vaud | Men | 53 | 52 | 48 | 40 | - 13 |
|  |  | Women | 17 | 20 | 21 | 20 | + 3 |
|  | Valais | Men | 56 | 55 | 47 | 37 | - 19 |
|  |  | Women | 14 | 17 | 18 | 18 | + 4 |

**Supplemental Figure S1:** Lifetime risk (in %) of lung cancer, by sex, per time-period, between 1995 and 2013, in Vaud and Valais, Switzerland (Source: Vaud and Valais Cancer Registries).

**Supplemental Table S3:** 10-year cumulative risk chart (in %) of lung cancer, for each age group, men (**A**) and women (**B**), per period, between 1995 and 2013, Vaud and Valais (Source: Vaud and Valais Cancer Registries).

| **A) MEN** |  |  |  |  |  | **B) WOMEN** |  |  |  |  |
| --- | --- | --- | --- | --- | --- | --- | --- | --- | --- | --- |
| **Age** | **1995-1998** | **1999-2003** | **2004-2008** | **2009-2013** |  | **Age** | **1995-1998** | **1999-2003** | **2004-2008** | **2009-2013** |
| **0** | 0.0 | 0.0 | 0.0 | 0.0 |  | **0** | 0.0 | 0.0 | 0.0 | 0.0 |
| **10** | 0.0 | 0.0 | 0.0 | 0.0 |  | **10** | 0.0 | 0.0 | 0.0 | 0.0 |
| **20** | 0.0 | 0.0 | 0.0 | 0.0 |  | **20** | 0.0 | 0.0 | 0.0 | 0.0 |
| **30** | 0.1 | 0.0 | 0.0 | 0.0 |  | **30** | 0.0 | 0.0 | 0.0 | 0.0 |
| **40** | 0.3 | 0.2 | 0.2 | 0.1 |  | **40** | 0.2 | 0.2 | 0.1 | 0.1 |
| **50** | 1.1 | 1.1 | 1.0 | 0.7 |  | **50** | 0.5 | 0.6 | 0.5 | 0.6 |
| **60** | 2.4 | 2.1 | 2.0 | 1.9 |  | **60** | 0.7 | 0.9 | 1.1 | 1.1 |
| **70** | 2.4 | 2.7 | 2.5 | 2.2 |  | **70** | 0.7 | 0.9 | 1.2 | 1.3 |
| **80** | 0.8 | 1.2 | 1.2 | 1.5 |  | **80** | 0.4 | 0.4 | 0.6 | 0.8 |
| **90** | 0.1 | 0.1 | 0.1 | 0.2 |  | **90** | 0.0 | 0.0 | 0.1 | 0.1 |
